# Thymic B cells in aging and autoimmune disease

**DOI:** 10.3389/fimmu.2025.1595805

**Published:** 2025-06-23

**Authors:** Sarah A. Wedemeyer, Ann V. Griffith

**Affiliations:** Department of Microbiology, Immunology, & Molecular Genetics, UT Health San Antonio, San Antonio, TX, United States

**Keywords:** thymus, aging, B cells, autoimmune disease, tolerance

## Abstract

Thymic B cells comprise a heterogenous population of cells localized primarily within the thymic medulla, a region populated by professional antigen-presenting cells (APCs) including dendritic cells, medullary thymic epithelial cells (mTECs), and macrophages. Through expression and presentation of self-antigens, these APCs are responsible for shaping the normal T cell repertoire by negatively selecting thymocytes recognizing self-antigens. It is now clear that thymic B cells have the capacity to participate in negative selection and present cognate antigens distinct from other medullary APCs, thus serving a non-redundant role in mediating T cell central tolerance. Recent work has linked thymic B cells with the development of multiple autoimmune diseases, many of which are increased in prevalence with aging. Here, we will provide a brief overview of the role of thymic B cell subsets in promoting negative selection and immune homeostasis, with a primary focus on the impact of aging on their tolerizing capacity and involvement in autoimmune diseases, highlighting thymic B cells as a potential novel therapeutic target to improve clinical outcomes in patients with autoimmune diseases.

## Introduction

Prior to Miller’s landmark discoveries highlighting the importance of the thymus in establishing protective immunity (1), investigators questioned whether the thymus was capable of producing antibodies as it had been observed in spleen and bone marrow (2). Early studies had not demonstrated antibody production from the thymus of immunized mice, suggesting a lack of antibody-producing plasma cells (3, 4). However, when Marshall and White introduced antigens directly into the thymus by intrathymic injection, they observed the accumulation of thymic antibody-secreting plasma cells and germinal centers, providing evidence of the existence of B cells in the thymus (5). These observations were supported by later studies describing plasma cells and germinal centers in the thymic medulla of chickens immunized with synovial fluid and liver and thymic protein extracts (6). The clinical significance of thymic B cells began to become more apparent with later studies noting the presence of antibody-producing thymic B cells in patients with the autoimmune disease myasthenia gravis, a chronic neuromuscular disease characterized by voluntary muscle weakness associated with difficulty swallowing, shortness of breath, ptosis, and impaired speech (7–10). As thymic hyperplasia and thymoma were known features of the disease, initial investigations led to the discovery of B cell clones in the thymus that produced acetylcholine receptor autoantibodies, as well as other striational autoantibodies with specificity for skeletal muscle (9). Immunohistochemistry studies revealed that thymic B cells localized in germinal centers in the thymus of myasthenia gravis patients, potentially contributing to the medullary hyperplasia seen in the disease (11). Thymic B cells were further implicated in disease through studies identifying the presence of B cell markers in mediastinal lymphomas suggested to be of thymic origin (12). However, it was unclear whether these B lymphocyte populations were native to the thymus and expanded during disease, or whether they infiltrated the thymus from the periphery to trigger disease onset.

Shortly after these clinical discoveries, it was revealed that thymic B cells were also present in small numbers (<1%) in the healthy adult thymus in both mice and humans (13, 14). The majority of these thymic B cells were found to belong to the B1 cell subset, which is a lineage of CD5-expressing, innate-like, IgM-secreting B cells with a distinct origin and functional properties from that of conventional B cells (14–16). Unlike conventional B cells, B1 cells are found in the peritoneal cavity but are not widely detected in the spleen, lymph nodes, or peripheral blood (14). Notably, B1 cells have been linked with the pathogenesis of multiple autoimmune diseases including lupus and rheumatoid arthritis (16, 17). Unlike thymic B cells of myasthenia gravis patients, which localize in organized lymphoid follicles in germinal centers, thymic B cells of healthy subjects were detected in the perivascular space of the medulla near Hassall’s corpuscles (18); and early histological studies noted they were found to associate with rosettes of T cells (19). Recent spatial imaging studies in the human infant thymus have further confirmed localization of thymic B cells in the medulla, forming niches with mTECs near Hassall’s corpuscles, suggesting they are poised to serve as APCs contributing to T cell tolerance induction (20, 21).

In recent years thymic B cells have indeed emerged as essential mediators of T cell negative selection, promoting tolerance to both neo-self antigens introduced in T cell receptor (TCR) transgenic systems as well as endogenous self-antigens in a polyclonal setting (22–25). Beyond presentation of B cell receptor (BCR)-captured antigens, thymic B cells can become licensed through CD40 stimulation to express Aire, a critical transcriptional regulator of tissue restricted antigen (TRA) gene expression, promoting the expression of TRAs distinct from those produced by mTECs (22). However, just as thymic aging is associated with significant declines in mTEC expression of Aire and Aire-associated TRAs (26–28), aging imparts significant disruptions in thymic B cell expression of Aire, which may reduce their tolerizing capacity (29). Supporting this notion, thymic B cells have been associated with the pathogenesis of numerous autoimmune diseases which have increased prevalence with aging, such as late-onset myasthenia gravis, Sjogren’s syndrome, and systemic lupus erythematosus (SLE) (30–35). In this review we will focus on aging-associated changes in thymic B cell function and their role in the development of autoimmune diseases from clinical studies and mouse models of autoimmunity.

## Heterogeneity of thymic B cell populations

Under homeostatic conditions, thymic B cells consist of a heterogenous population of cells in both mice and humans, including medullary B cells involved in antigen presentation, as well as a population of perivascular memory B cells and plasma cells (PCs), each with diverse functions contributing to immune homeostasis (18). Thymic PCs specific to common viral antigens accumulate in human thymus during the first year of life, and constitutively secrete complement-fixing IgG1 and IgG3 (36). Localized within the perivascular space, these PCs may serve an important antimicrobial role in the thymus to protect against infection-induced pathology.

Under healthy conditions in both mice (25, 37) and in humans (19, 38), a large percentage of thymic B cells have undergone class-switching. In C57BL/6 mice, IgM^-^ IgD^-^ isotype-switched thymic B cells are predominantly composed of IgG2b^+^, IgA^+^, IgG2c^+^, and IgG1^+^ B cells (37). Analysis of AID expression and circle transcripts suggests that class switching occurs intrathymically under steady-state conditions and is dependent upon TCR/CD40 signaling with mature thymocytes. Importantly, the BCR repertoire of class-switched thymic B cells is distinct from that of non-class-switched B cells and is particularly skewed toward self-antigens (37). These findings were confirmed in a later study by Lombard et. al., in which thymic B cell isotype switching was evaluated among a variety of common mouse strains (C3H, A/J, NZO) and wild-derived inbred strains (PWK, CAST) (25). Among these strains, the most common isotype-switched thymic B cells expressed an IgG or IgA BCR (25). Castaneda and colleagues have also recently noted the presence of class-switched memory B cells in the murine thymus that are detectable during the neonatal period, before the emergence of peripheral class-switched B cells (39). Importantly, thymic memory B cell differentiation was not driven by foreign antigen stimulation, but instead depended upon interactions with CD4^+^ T cells, supporting a role as local antigen-presenting cells to establish T cell central tolerance as discussed below. Similarly, thymic B cells sorted from pediatric patients displayed a mature phenotype and were enriched for protein autoantigen binding, further implicating a role of thymic B cells in the presentation of autoantigens to establish T cell central tolerance (38).

In contrast with splenic B cells, thymic B cells largely display an activated, mature phenotype and express higher levels of MHC class II and costimulatory molecules CD80/86, suggesting they are functionally different from other peripheral B cell populations and are poised to interact with T cells in the thymus (22, 40, 41). Thymic B cell CD40-CD40L interactions with T cells appears to be a particularly strong requirement for their survival and proliferation, as mice lacking CD40L demonstrate a severe reduction in the total number of thymic B cells (42, 43). Further, autonomous expression of CD40 by thymic B cells is required to support their overall population maintenance (43) as well as promote high efficiency antigen presentation to T cells (41). In contrast, expression of neither MHC class II nor CD80/86 is required for thymic B cell maintenance, highlighting the unique role of CD40 in regulating the thymic B cell population (43).

## Role of thymic B cells in mediating central tolerance

Evidence for the capacity of thymic B cells to mediate clonal deletion of T cells was first shown in deletion of viral superantigens, which bind outside the antigen-binding groove of major histocompatibility complex (MHC) class II molecules, resulting in excessive stimulation of the host T cells (44). Intrathymically injecting thymic B cells, but not dendritic cells or splenic B cells, results in the clonal deletion of Mls-1-specific T cells in neonatal BALB/c mice, and induced tolerance in a graft vs. host assay, while injection of dendritic cells promotes anergy of Mls-1-reactive T cells rather than deletion,suggesting that thymic B cells were the main mediators of clonal deletion of superantigen-specific T cells (45).

In 2015 Yamano et al. demonstrated that thymic B cells, upon interaction with CD4 single-positive (SP) thymocytes in the context of CD40 signaling, acquire tolerogenic features, such as expression of Aire, and induced transcription of unique TRAs expressed in the brain (*Grik2*), spermatocytes (*Ggn*), and lung (*Lamp3*) that were weakly expressed in mTECs (22). Notably, mature peripheral B cells were able to readily acquire this licensing phenotype when exposed to the thymic microenvironment. They found that the CD4 SP compartment was significantly larger in B cell-deficient *Mb1*-Cre knock-in mice relative to controls, further demonstrating the importance of thymic B cells in promoting negative selection of CD4 SP T cells (22).

Following these studies confirming a role for thymic B cells in promoting T cell tolerance induction, thymic B cell-mediated negative selection of certain neo-self-antigens was subsequently found to be dependent upon CD40-dependent Aire-licensing (22). Yamano et al. utilized an Aire-HCO transgenic model in which a chimeric influenza hemagglutinin (HA) protein is expressed by both mTECs and thymic B cells licensed to express Aire (22). After co-culturing purified thymic B cells, DCs, mTECs, and peripheral B cells with GFP-expressing HA-specific A5 hybridoma T cells, they found that mTECs strongly stimulated the A5 cells, while both thymic B cells and DCs both presented HA with similar efficacy. However, by transplanting Aire-HCO bone marrow into wild-type (WT) recipients, they found that HA presentation by thymic B cells remained intact, confirming that thymic B cells directly present endogenously expressed antigen.

The capacity of thymic B cells to participate in the negative selection of autoreactive CD4 T cells has been thoroughly investigated using other neo-antigen systems also. Frommer et al. utilized a B^MOG^ mouse line crossed with 2D2 mice (which express a MOG_p35-55_-specific TCR) to examine the role of B cell-specific presentation of MOG (Myelin Oligodendrocyte Glycoprotein) peptide to T cells via MHC class II (23). They found that the number of CD4 SP T cells in the thymi and lymph nodes of these B^MOG^/2D2 mice was significantly reduced compared to 2D2 control mice. Importantly, crossing these mice with B cell-deficient JHT mice revealed that presentation of MOG peptide was unique to thymic B cells, and not performed by other APCs (including DCs, macrophages, and mTECs). This negative selection of MOG-specific T cells was sufficient to prevent the induction of experimental autoimmune encephalitis (EAE) in B^MOG^-2D2 mice. In a subsequent study, Perera et al. crossed 121 BCR knock-in mice specific for glucose-6-phosphate isomerase (GPI) with KRN TCR transgenic mice (which recognize GPI_282–292_ peptide presented by MHC class II). They observed a three-fold reduction in the number of CD4 SP T cells and overall reduced thymic cellularity compared to controls (24), providing further evidence that despite their relatively low frequency, thymic B cells serve as efficient antigen-presenting cells for T cell negative selection.

Thymic B cells have also been shown to mediate tolerance to a group of Aire-independent autoantigens. Afzali et al. recently demonstrated that B cells endogenously express AQP4, an autoantigen linked with neuromyelitis optica, in response to CD40 stimulation (46). These AQP4-expressing thymic B cells promote efficient negative selection of AQP4-TCR^+^ CD4 T cells. Notably, B cell-specific deletion of *Aqp4* resulted in a rescue of AQP4-TCR^+^ T cells (despite AQP4 expression by mTECs), highlighting the unique role of thymic B cells in establishing tolerance to this autoantigen.

Recently, Lombard et al. demonstrated the capacity of thymic B cells to promote negative selection of CD4 SP T cells in a polyclonal setting, which is dependent upon AID expression (25) (reviewed in (47)). Using NOD (non-obese diabetic) mice crossed with AID^-/-^ mice, which lack class switch recombination in thymic B cells, they observed that AID deficiency was associated with an accelerated development of diabetes onset compared to NOD.AID^+/+^ littermates. They also noted an enlargement of the spleen and pancreatic lymph nodes with a coinciding increase in the frequency and number of activated (CD69^+^) CD4 T cells. Notably, NOD.Rag^-/-^ mice (lacking B and T cells) receiving total thymocytes from NOD.AID^-/-^ mice had significantly more insulitis compared to mice receiving thymocytes from NOD.AID^+/+^ mice. Further, through cleaved caspase-3 staining, they observed that fewer CD4 SP thymocytes were found to be undergoing clonal deletion in NOD.AID^-/-^ mice compared to NOD.AID^+/+^ controls, suggesting impaired negative selection in AID-deficient NOD mice. Next, to determine the role of AID expression by thymic B cells in the negative selection of a neo-self antigen, they crossed AID^-/-^ mice with 3A9 TCR transgenic mice, which express a TCR specific for a peptide from HEL (hen egg lysozyme) protein. They found a 20% increase in thymic 3A9^+^ CD4 SP T cells from AID^-/-^ mice compared to AID^+/+^ controls, demonstrating an important role for AID expression by thymic B cells in promoting efficient negative selection of T cells.

Thymic B cells are also implicated in promoting T cell negative selection in humans. The infant thymus was found to have heterogenous populations of CD19^+^ thymic B cells, including a significant (approximately 50%) population of CD21^-/low^ memory B cells localized in the medulla that express high levels of Aire and activation markers CD69, CD95, and CD86 (48). Subsequently, evaluation of the human thymic B cell transcriptome revealed significant expression of *CDH17* and *LAMP3*—representative TRAs of the intestinal tract and the lung, respectively—concomitantly with a lower expression of *KLRB1*, an Aire-repressed gene, in thymic B cells (49). Further, RNA seq analysis on sorted human B cells revealed expression of TRAs associated with autoimmune diseases, such as LAD1 (linear IgA dermatosis), SCG2 (autoimmune hypophysitis), ICA1 (type 1 diabetes), ACHE and AKAP12 (myasthenia gravis), and METRNL (autoimmune hepatitis) (49), implicating a potential role for thymic B cells in establishment of self-tolerance towards a variety of peripheral tissues.

In addition to establishing central tolerance through mediating negative selection of autoreactive thymocytes, thymic B cells have been suggested to sizably contribute to the maintenance of thymic regulatory T (Treg) cells (50). Used as a mouse model of lupus and Sjogren’s syndrome, BAFF-overexpressing mice (BAFF-Tg) have increased numbers of peripheral Foxp3^+^ CD4 Tregs with concomitant expansion of B2 cells, which is associated with the development of autoimmunity (51). This expansion of peripheral Tregs was found to be due to increased generation of thymus-derived Helios^+^ Foxp3^+^ Tregs (52). To determine the necessity of B cells in promoting this expansion of thymic Foxp3^+^ Tregs, Walters et al. generated bone marrow chimeras consisting of WT or BAFF-Tg hosts transplanted with WT or μMT^-/-^ (B cell deficient) bone marrow (52). In the absence of mature B cells, there was a significant reduction in the frequency of CD4^+^ Foxp3^+^ thymic Tregs even in the presence of BAFF (52). μMT^-/-^ mice also have significantly reduced frequency and total number of CD4^+^ Foxp3^+^ positive thymic Tregs compared to WT controls (52). Furthermore, limiting BCR repertoire diversity resulted in a reduction in the frequency of thymic Tregs, suggesting a diverse BCR repertoire is required for thymic B cells to fully participate in antigen capture and subsequent presentation to nascent T cells (52). An association between thymic B cells and thymic Treg generation was further confirmed using a μMT^-/-^ Foxp3-GFP reporter system (40). Lu et al. demonstrated co-localization of thymic B cells with thymic Tregs in the medulla through immunofluorescence microscopy, suggesting potential T cell divergence to the Treg lineage via direct contact with thymic B cells. Notably, T cells co-cultured with thymic B cells were able to develop into Foxp3^+^ Tregs, and this development was blocked upon MHC class II blockade (40). Advancing the notion that thymic B cells are involved in generation of thymic Tregs, Martinez et al. demonstrated the contribution of low-grade “sterile” inflammation via type III interferon signaling in promoting the activation of thymic B cells and class switch recombination leading to the generation of thymic Tregs (53).

Recent fate mapping studies have demonstrated that thymic B cells also function in concert with specialized mimetic subsets of TEC, such as microfoldTEC, to promote their maturation and enable antigen transfer to thymic APCs (54). In turn, these interactions promote secretion of TNF superfamily member APRIL from nearby CX3CR1^+^ thymic APCs, promoting differentiation of thymic B cells into class-switched IgA^+^ plasma cells, revealing a novel cross-talk mechanism that may further drive the tolerogenic and/or antimicrobial functions of this specialized population.

Thus, growing evidence suggests that thymic B cells help mediate T cell central tolerance by promoting clonal deletion of autoreactive thymocytes, by diverting potentially autoreactive thymocytes to a regulatory T cell lineage, and by promoting the tolerizing functions of mTECs.

## Impact of aging on thymic B cell function

Aging of the adaptive immune system leads to both impaired responses to new infections and vaccinations, as well as a dysregulation of self-tolerance leading to autoimmunity and inflammatory disease (55). Within the B cell compartment, aging is associated with an expansion of a population of unique CD11c^+^, T-bet^+^ peripheral B cells, designated ABCs (age-associated B cells), within the spleen and bone marrow (56–59). Outside the expansion seen during aging, proliferation of ABCs has also been observed in both mice and in humans during chronic viral infection and in autoimmune conditions including SLE (60, 61). ABCs, unlike other memory B cells, do not respond to BCR or CD40 stimulation but instead respond to TLR9 and TLR7, secreting cytokines such as IL-4 and IL-10 as well as autoantibodies (56, 62). Expression of T-bet, a transcription factor expressed by T cells to promote Th1 differentiation, is required for the production of autoantibodies by ABCs as well as IgG2a class switching (62). Patients with lupus were found to have a population of ABCs capable of differentiating into plasma cells that produced autoantibodies (63). In addition to their direct role in autoantibody production contributing to autoimmunity, a subset of aged B1 B cells were reported to accumulate in adipose tissue of mice in the context of sepsis, which is associated with increased pro-inflammatory macrophages and compromised lipolysis (64). Conversely, μMT^-/-^ mice were found to have improved adipocyte metabolism and reduced NLRP3 inflammasome activity by pro-inflammatory macrophages, highlighting the interactions between aged B cells and macrophages driving impaired lipid metabolism in WAT driving inflammation during sepsis (64).

**Figure 1:**
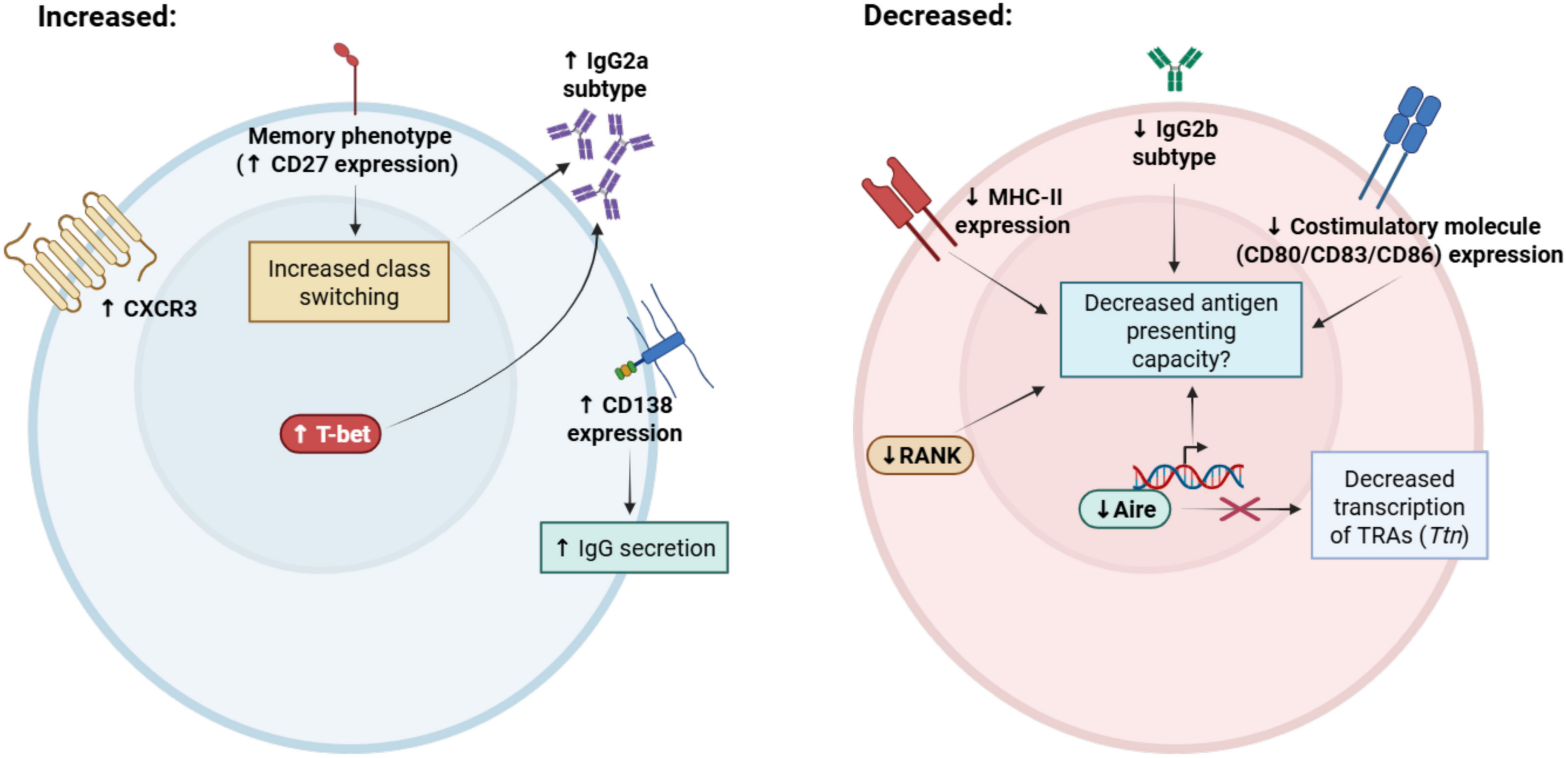
Summary of phenotypic alterations associated with aging in thymic B cells. Thymic B cells acquire distinct phenotypic changes with aging that may contribute to age-associated declines in function. Aged thymic B cells have increased expression of memory markers (such as CD27 and CXCR3) and significantly reduced expression of MHC class II and costimulatory molecules CD80/CD83/CD86. IgG2a class-switching is increased and IgG2b class-switching is decreased with age, which is associated with increased expression of T-bet and increased frequency of the age-associated B cell (ABC) phenotype. Notably, expression of the ABC marker CD11c is not increased with age in thymic B cells. Aged thymic B cells have decreased expression of receptor activator of NF-κB (RANK), which promotes mTEC maturation and is a positive regulator of Aire expression. Despite maintained CD40 expression, which is required for thymic B cell Aire licensing, Aire expression declines significantly, with the largest reductions occurring in IgG class-switched B cells. Expression of Aire-dependent and Aire-independent tissue-restricted antigens are also decreased with aging, including Titin (*Ttn*), which is associated with the development of late-onset myasthenia gravis. Taken together, these alterations may impair antigen-presenting capacity in thymic B cells, resulting in age-associated declines in T cell central tolerance induction. Figure was generated in BioRender.com.

In the thymus there is an increased relative frequency of CD19^+^ B cells in C57BL/6 mice during aging, despite decreased B cell potential of early thymic progenitors (ETPs) in adult mice relative to neonates (29, 65). We estimate that the frequency of thymic B cells increases nearly sixfold, from 0.2% to 1.2% of all thymocytes by one year of age in mice, while in humans, the relative frequency of thymic B cells is estimated to be higher, increasing from <5% in infants to 5-15% in adults (36). Despite an increased frequency, the total number of thymic B cells in aged mice remains unchanged, due to age-associated declines in overall thymus cellularity (29, 66). Beyond changes in their relative frequency, we reported that aged thymic B cells display phenotypic changes in mice (summarized in **Figure 1**), including loss of Aire expression and decreased expression of tissue-restricted antigens, including Titin (*Ttn*), an important antigen linked with development of late-onset myasthenia gravis (29, 67). Aged thymic B cells were also found to have increased IgG2a frequency and decreased IgG2b frequency (29), which is consistent with increased ABC frequency and T-bet expression with age (58, 68). Interestingly, aged thymic B cells were found to have reduced expression of RANK, but not CD40, which is required for licensing of Aire expression in young thymic B cells and for overall maintenance of the thymic B cell pool (22, 43). In contrast, while peripheral ABCs are characterized by increased CD11c expression (57), thymic B cells from aged mice were found to have no significant increase in CD11c expression (29), suggesting phenotypic differences between aged thymic B cells and peripheral ABCs. While decreased expression of Aire by aged thymic B cells is speculated to diminish their capacity to mediate negative selection leading to the development of autoimmunity (29), further studies will be required to mechanistically link aging thymic B cell phenotypic alterations with loss of self-tolerance.

Recent histological and transcriptomic studies have also confirmed age-associated changes in thymic B cell subset frequency and phenotype in the human thymus. Investigating phenotypic changes in human thymic B cell populations across the lifespan, Nunez et al. demonstrated there was a nearly 10-fold reduction in the expression of MHC class II inadult thymic B cells compared to infant thymic B cells, as well as a significant reduction in the levels of co-stimulatory molecules CD80, CD83, and CD86 with aging (36). In addition, expression of the chemokine receptor CXCR3 was significantly increased in thymic B cells from older adults, suggesting these B cells acquire a memory phenotype. CD27^+^ class-switched memory B cells steadily increase in frequency after the first year of life, consistent with the accumulation of memory B cells throughout aging (49). In agreement with these observations, Park et al. observed an increase in the frequency of thymic memory B and T cells in adult thymus relative to infant thymus during aging (69). Thus, aging induces significant alterations in the thymic B cell repertoire of both mice and humans, which may significantly impact their tolerogenic function. We will next discuss the association of thymic B cells with various autoimmune conditions, many of which are increased in frequency with aging.

## Contribution of thymic B cells to autoimmunity

Thymic B cells have been associated with numerous autoimmune diseases, while the exact role these cells play in the prevention and/or progression of these conditions has been incompletely understood. B cell-targeting therapies are increasingly being tested in Phase 1/2 clinical trials to treat numerous refractory autoimmune diseases, including systemic lupus erythematosus (SLE), multiple sclerosis, and myasthenia gravis (70, 71). These approaches include anti-CD19 monoclonal antibody (Rituximab) treatment, chimeric antigen receptor (CAR)-T cell therapy, and anti-BAFF monoclonal antibody (Belimumab) therapies (72–76). While these therapies are expected to target activation of peripheral CD19-expressing B cells, the potential impact of these treatments on thymic B cell function should also be considered. In this section we will present an overview of what is currently known about the contribution of thymic B cells to autoimmunity from both human clinical studies and animal models of disease.

## Myasthenia gravis

In 1936, American surgeon Alfred Blalock pioneered treatment for myasthenia gravis by removing a thymic tumor (77). Blalock hypothesized that thymic pathology played a role in contributing to the disease, and promoted thymectomy as a treatment for myasthenia gravis even in the absence of a thymic tumor (nonthymomatous myasthenia gravis). Support for thymectomy as a treatment for myasthenia gravis has been further boosted by recent clinical trials demonstrating improvements in myasthenia gravis symptoms (78, 79). Anti-acetylcholine receptor (AChR) autoantibody-producing B cells have since been discovered in the thymus of myasthenia gravis patients, suggesting their involvement in disease pathogenesis (8, 30, 80). Histological studies have noted significantthymic architectural disruption in myasthenia gravis patients characterized by deformation of the medullary epithelial compartment and expansion of interlobular and perivascular spaces containing numerous secondary follicles with IgM- and IgD- expressing B cells (81). Thymic B cells are expanded in myasthenia gravis patients relative to healthy subjects, and phenotyping studies have confirmed they possess an activated phenotype (30, 31). Isolated thymic lymphocytes from myasthenia gravis patients were found to be capable of spontaneously secreting IgG, IgM, and IgA, supporting a role for B cell activation in the progression of disease (82).

While nearly 90% of myasthenia gravis patients have autoantibodies targeting AChR and/or the muscle-specific tyrosine kinase (MuSK), other autoantibodies targeting striated muscles, particularly the sarcomere protein Titin, have been detected in some myasthenia gravis patients. Anti-Titin autoantibodies have been detected in a significant (80%) number of patients with thymomatous myasthenia gravis, while they are present only in a minority of myasthenia gravis patients without thymoma (83). The presence of anti-Titin autoantibodies is a distinctive feature of late-onset (>50 years) myasthenia gravis, with nearly 50% of patients diagnosed with late-onset myasthenia gravis having detectable anti-Titin autoantibodies, while anti-Titin autoantibodies are rare in patients with early-onset disease (84). Anti-Titin autoantibodies have also been detected in a majority of late-onset myasthenia gravis patients with myopathy, suggesting aging-associated perturbations contribute to this increased susceptibility (85). We have previously reported a decrease in thymic B cell expression of *Ttn* with aging in mice (29). Whether this decreased expression promotes a breakdown in T cell tolerance against Titin and increased susceptibility to myasthenia gravis warrants further investigation. Thus, thymic B cells play a prominent role in mediating both early-onset and late-onset myasthenia gravis pathogenesis through potentially distinct mechanisms, making them a promising therapeutic target for disease intervention.

## Neuromyelitis optica

Neuromyelitis optica is an autoimmune disease affecting the central nervous system driven primarily by autoantibodies against the water channel protein AQP4. Astrocytes primarily express the M23 isoform (86), which is a prime target against anti-AQP4 autoantibodies. Because these anti-AQP4 antibodies are class-switched, this suggests they occur following a germinal center (GC) reaction, which requires an antigen-specific T follicular helper cell response. Afzali et al. recently hypothesized that B cells play an important role in preventing T cell- B cell mediated GC reactions that result in autoantibody production (46). They noted that while both mTECs and thymic B cells endogenously express AQP4, anti-CD40 and IL-21 stimulation promoted the expression of AQP4 in thymic B cells. Through genetic ablation of *Aqp4* in B cells, they found that thymic B cells were a significant contributor to the negative selection of AQP4-specific T cells (46). In contrast, genetic ablation of *Aqp4* in TECs did not affect total numbers of AQP4-specific T cells. In a model of experimental autoimmune encephalitis (EAE), they noted that B cell-specific *Aqp4* knock-out mice were significantly more susceptible to clinical disease induced by immunization with AQP4_p41_ peptide compared to mTEC-specific *Aqp4* knock-out mice. These results suggest that even though mTECs endogenously express AQP4, thymic B cell- T cell interactions are critical for shaping tolerance against AQP4-reactive T cells to prevent GC reactions leading to autoantibody production.

## Systemic lupus erythematosus

While peripheral B cells are well-established as drivers of lupus pathogenesis via promoting antibody production, T cell activation, and cytokine secretion (87), thymic B cells may also play a significant role in SLE pathogenesis. Previous histological studies noted that in comparison to healthy patients and patients with myasthenia gravis, thymic samples from patients with SLE were found to have distinct architectural adaptations, including the presence of epithelial aggregates in the medulla, cortical thinning, GC formation, and increased plasma cells (88, 89). There are limited human studies investigating the role of thymic pathology in the development of SLE, but a variety of environmental and genetic mouse models suggest an association between thymic B cells and SLE incidence. Increased thymic B cell frequency has been observed in MRL/lpr lupus-prone mice, along with increased frequencies and total numbers of CD4 SP and CD8 SP T cells, suggesting reduced negative selection (90). Likewise, lupus-prone NZBWF1/J (BWF1) mice, which develop spontaneous autoimmunity resembling SLE, have a greater than 20-fold increase in B cell frequency and a 6-fold increase in absolute B cell numbers in the thymus compared with age-matched control mice (32). Further, thymic B cells in BWF1 mice proliferated and clustered in structures resembling ectopic GCs. Similarly, using the pristane-induced model of lupus, Tang et al. found a substantial increase in the frequency of thymic B cells, suggesting genetic as well as pharmacological models of SLE are linked with an accumulation of thymic B cells (33). Thus, it is possible that the thymus, by housing its own population of B cells and plasma cells that accumulates with aging, can become a specialized niche that promotes autoreactive humoral responses associated with SLE (32).

## Type 1 diabetes

T1D development is associated with a breakdown in T cell central tolerance leading to destruction of the insulin-producing pancreatic beta cells. Previous studies with non-obese diabetic (NOD) mice noted a gradual accumulation of thymic B cells and concomitant loss of mTECs throughout progression of disease (91, 92). Following up on these initial findings, thymic B cells were found to develop abnormally during the late insulitic-prediabetic phase in NOD mice, suggesting a potential contribution of thymic B cells to defects in T cell central tolerance (93) (reviewed in (94)). Ectopic germinal centers and increased numbers of class-switched IgD^+^ and IgE^+^ B cells have been detected in the thymi of NOD mice compared to controls (93). However, absolute numbers of insulin-reactive B cells in the thymus are similar between NOD mice and C57BL/6 control mice, suggesting that the number of insulin-reactive thymic B cells does not correlate with T1D susceptibility. In contrast, NOD mice demonstrated binding of mouse immunoglobulins to mTECs, which was associated with increased apoptosis of insulin-expressing mTECs (93). NOD thymocytes cultured in the presence of whole insulin or proinsulin peptide with bone marrow-derived DCs were found to be significantly more responsive relative to thymocytes from B cell-deficient NOD-μMT^-/-^ mice. Overall, these results suggest that autoreactive thymic B cells target insulin-expressing mTECs, which allows escape of insulin-reactive T cells driving T1D susceptibility (93).

A recent study from Lombard et al. provides further mechanistic insight linking thymic B cells with T1D pathogenesis (25). The authors noted decreased expression of the costimulatory molecule CD80 in thymic B cells, but not other thymic APCs, in NOD mice relative to C57BL/6 mice. This decreased costimulatory molecule expression resulted in inefficient thymic B cell activation and differentiation into isotype-switched B cells which are associated with promoting T cell central tolerance (25). Further suggesting impaired negative selection, the authors noted a greater frequency and total number of CD4 SP T cells in NOD mice relative to controls (25). To investigate the importance of thymic B cell class switching in thymocyte selection and tolerance, NOD.AID^-/-^ mice were generated, and were found to have accelerated onset of diabetes relative to NOD.AID^+/+^ mice with a concomitant increase in autoreactive CD4 T cells. These results, which support a role for AID expression in regulating B cell class switch recombination and subsequent T cell negative selection, are in agreement with previous clinical studies which linked AID mutations with peripheral organ autoimmunity including diabetes in patients with hyper-IgM syndrome (95). Therefore, thymic B cell expression of AID plays a nonredundant role in inducing CD4^+^ T cell tolerance controlling autoimmune disease susceptibility.

## Sjogren’s syndrome

Two mouse models of Sjogren’s syndrome, an autoimmune disease characterized by autoantibody-mediated inflammation of the salivary and lacrimal glands driven by B cell hyperactivity, have been noted to have distinct thymic pathology (96). IQI/*Jic* mice spontaneously develop focal lymphocytic inflammation after 6 months of age in the lacrimal and salivary glands with a coinciding increase in thymic B cells in aged females (34, 35). The Aly/*aly* mouse carries the homozygous autosomal recessive alymphoplasia (*aly*) gene mutation and is marked by a systemic absence of lymph nodes and Peyer’s patches as well as disorganized spleen and thymic architecture characterized by a sparse medulla and enlarged cortex with a reduction in mTECs leading to disrupted cortical-medullary boundaries (97, 98). Several clinical case reports have noted the development of thymic B cell mucosa-associated lymphoid tissue (MALT) lymphomas arising in patients with pre-existing Sjogren’s syndrome (99–101) and/or salivary B cell MALT lymphoma (102), suggesting thymic B cells may be a driver of these pathologies. Histological analysis of thymic B cell MALT lymphomas revealed dense B lymphocyte infiltration into the Hassall’s corpuscles forming lymphoepithelial lesions resembling myoepithelial sialadenitis (MESA) lesions observed in Sjogren’s syndrome (103), further implicating thymic B cells in the development of salivary gland autoimmunity and/or MALT lymphoma. These observations suggest the possibility that chronic inflammation in the thymus driven by autoreactive thymic B cells linked with Sjogren’s syndrome pathology plays a role in the development of subsequent thymic B cell MALT lymphomas.

## Food allergy

Food allergies are characterized by IgE-mediated hypersensitivity reactions triggering mast cell activation and subsequent clinical symptoms ranging from mild skin reactions to potentially life-threatening anaphylaxis (104). As the thymus has been found to contain a reservoir of antibody-secreting plasma cells maintained throughout the lifespan (105), it was speculated that the thymus may be a source of IgG and/or IgE-secreting plasma cells specific for dietary antigens involved in food allergy. Recently, plasma cells secreting IgG specific to common cow’s milk antigens (alpha-lactalbumin, lactoferrin, and casein) were detected in the human infant thymus (106). In A/J mice, which are commonly used to study environmental carcinogens in tumorigenesis, a population of IgE-secreting cells in the thymus was found to develop after the first postnatal week, and this population induced T cell tolerance to IgE within the first few weeks of life (107). A similar population of IgE-producing cells was noted in BALB/c mice (107). In BALB/c mice Kwon et al. noted a significant population of IgE-secreting CD138^+^ Blimp1^+^ plasma cells in the thymus induced by IL-4 signaling from iNKT cells (108). In this study, thymus-derived IgEs were found to expand the frequency of mast cells in the gut and correlated with the severity of food anaphylaxis induction. After thymectomy, serum IgE levels and severity of food anaphylaxis declined significantly. Thus, IgE-producing thymic plasma cells have been associated with both tolerance and exacerbation of pathology in allergic disorders.

## Conclusions/future directions

**Figure 2:**
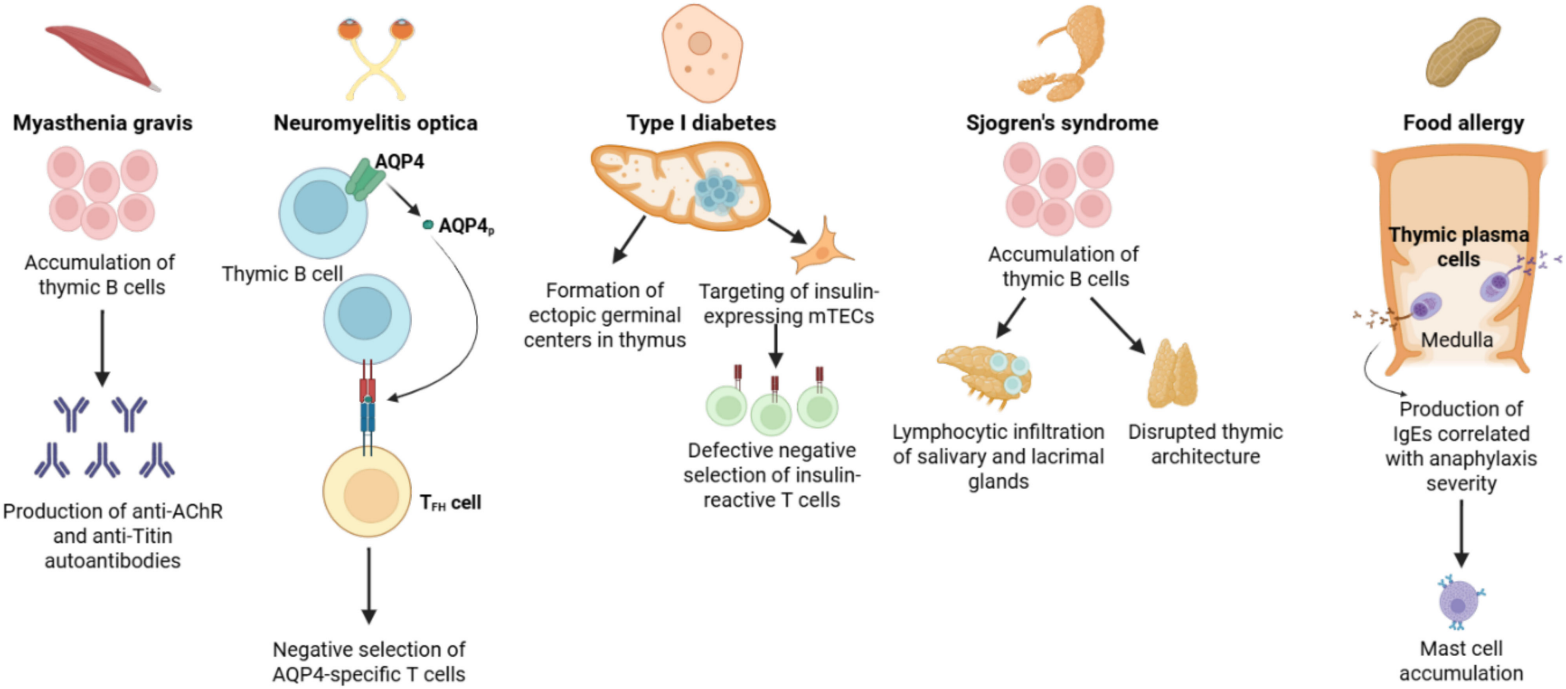
Overview of the contribution of thymic B cells to autoimmune disease pathogenesis. Thymic B cells mediate induction of central tolerance against a variety of self-antigens and are thus implicated in the induction of several autoimmune diseases. Myasthenia gravis is associated with an accumulation of thymic B cells which can produce autoantibodies against the acetylcholine receptor (AChR) and the muscle sarcomere protein Titin. Thymic B cell expression of the water channel AQP4 has been shown to be required for the negative selection of AQP4-reactive T cells, preventing T_FH_ cell interactions with B cells driving neuromyelitis optica. Autoimmune diseases such as SLE and type I diabetes (T1D) are associated with an accumulation of thymic B cells and the formation of ectopic germinal centers. In T1D, this is associated with the targeting and destruction of insulin-expressing mTECs, resulting in impaired negative selection of insulin-reactive T cells. An accumulation of thymic B cells may disrupt thymic architecture and trigger lymphocytic infiltration of the salivary and lacrimal glands leading to development of Sjogren’s syndrome. A subgroup of IgE-secreting thymic plasma cells is present under homeostatic conditions, promoting expansion of mast cells in the skin and gut, which is correlated with the severity of anaphylaxis induction in mice and may be linked with development of food allergy. Figure was generated in BioRender.com.

Despite older individuals experiencing a significantly higher incidence of autoimmune disease (109), the precise mechanisms driving this increased susceptibility remain incompletely resolved. Thymic B cells have emerged as critical, nonredundant mediators of T cell self-tolerance through both BCR-acquired antigen and expression and presentation of Aire-dependent and Aire-independent tissue-restricted antigen genes unique from those expressed by mTECs and presented by other thymic APCs (22–24). However, the thymus is among the most rapidly aging tissues in the body and undergoes pronounced atrophy with age-associated declines in APC function (reviewed in (66)). It is now understood that thymic B cells also undergo phenotypic alterations with aging, including increased expression of the transcription factor T-bet and IgG2a class-switching associated with ABCs, as well as loss of Aire and costimulatory molecule expression, all of which may contribute to age-associated loss of tolerance against antigens presented by thymic B cells (29). Supporting this notion, several autoimmune diseases with age-associated increases in incidence, are associated with thymic B cell abnormalities, including late-onset myasthenia gravis, systemic lupus erythematosus, Sjogren’s syndrome, and neuromyelitis optica (summarized in **Figure 2**). The clinical implications of thymus-resident B cells and plasma cells shaping systemic tolerance to common environmental and dietary antigens are especially striking and warrant further investigation, as effective strategies to mitigate IgE-mediated food allergies are lacking despite sharply increasing prevalence of food allergies among children and adults in the US population (110). Thus, there is a critical need to more comprehensively define the contribution of thymic B cells to negative selection under steady-state, aging, and autoimmune conditions to inform the design of therapeutic approaches to restore and maintain T cell tolerance.
